# The *Operophtera brumata* Nucleopolyhedrovirus (OpbuNPV) Represents an Early, Divergent Lineage within Genus *Alphabaculovirus*

**DOI:** 10.3390/v9100307

**Published:** 2017-10-21

**Authors:** Robert L. Harrison, Daniel L. Rowley, Joseph D. Mowery, Gary R. Bauchan, John P. Burand

**Affiliations:** 1 Invasive Insect Biocontrol and Behavior Laboratory, Beltsville Agricultural Research Center, USDA Agricultural Research Service, Beltsville, MD 20705, USA; Daniel.Rowley@ars.usda.gov; 2 Electron and Confocal Microscopy Unit, Beltsville Agricultural Research Center, USDA Agricultural Research Service, Beltsville, MD 20705, USA; Joseph.Mowery@ars.usda.gov (J.D.M.); Gary.Bauchan@ars.usda.gov (G.R.B.); 3 Department of Microbiology, University of Massachusetts-Amherst, Amherst, MA 01003, USA; jburand@microbio.umass.edu

**Keywords:** baculovirus, *Alphabaculovirus*, genome, winter moth, *Operophtera brumata*, cypovirus

## Abstract

Operophtera brumata nucleopolyhedrovirus (OpbuNPV) infects the larvae of the winter moth, *Operophtera brumata*. As part of an effort to explore the pesticidal potential of OpbuNPV, an isolate of this virus from Massachusetts (USA)—OpbuNPV-MA—was characterized by electron microscopy of OpbuNPV occlusion bodies (OBs) and by sequencing of the viral genome. The OBs of OpbuNPV-MA consisted of irregular polyhedra and contained virions consisting of a single rod-shaped nucleocapsid within each envelope. Presumptive cypovirus OBs were also detected in sections of the OB preparation. The OpbuNPV-MA genome assembly yielded a circular contig of 119,054 bp and was found to contain little genetic variation, with most polymorphisms occurring at a frequency of < 6%. A total of 130 open reading frames (ORFs) were annotated, including the 38 core genes of *Baculoviridae*, along with five homologous repeat (*hr*) regions. The results of BLASTp and phylogenetic analysis with selected ORFs indicated that OpbuNPV-MA is not closely related to other alphabaculoviruses. Phylogenies based on concatenated core gene amino acid sequence alignments placed OpbuNPV-MA on a basal branch lying outside other alphabaculovirus clades. These results indicate that OpbuNPV-MA represents a divergent baculovirus lineage that appeared early during the diversification of genus *Alphabaculovirus*.

## 1. Introduction

Baculoviruses are rod-shaped, insect-specific viruses of family *Baculoviridae* that possess large (≥80 kbp) double-stranded circular DNA genomes [1]. There are four genera in this family, with genus *Alphabaculovirus* containing the largest number of species. Alphabaculoviruses—also known as nucleopolyhedroviruses (NPVs)—infect larvae of order Lepidoptera (moths and butterflies) and produce visually distinctive polyhedra (or occlusion bodies, OBs) in host cells during replication [2,3]. The OBs are large enough to be visualized by light microscopy, and contain a type of virion referred to as the occlusion-derived virus (ODV). The ODV initiate primary infection of the host larval midgut epithelium after being liberated from the OBs, which are solubilized in the alkaline lumen of the host midgut. A second type of virion—the budded virus (BV)—is initially assembled and secreted from infected cells to spread infection to other tissues in the host. Progeny ODVs are later assembled and occluded into OBs, which are subsequently released after the death of the infected insect.

In this study, we present an analysis of the occlusion bodies and genome sequence of an alphabaculovirus from the winter moth, *Operophtera brumata* (L.) (Lepidoptera: Geometridae). A native of Europe, this moth species has established invasive populations in North America multiple times during the last 60 years [4,5]. The most recent invasion started in Massachusetts, and has spread to the coastal regions of New England in the USA and the Maritime Provinces in Canada. Outbreaks of winter moth larvae in these areas cause mass defoliation of trees. The larvae also attack the fruiting buds of apple trees and blueberry bushes in local orchards.

Alphabaculovirus isolates have been isolated from populations of winter moth [6,7], including winter moth larvae in Massachusetts [8]. As a pathogen of the winter moth, the idea of formulating Operophtera brumata nucleopolyhedrovirus (OpbuNPV) isolates as biopesticides to use against outbreaks of winter moth larvae is appealing. Another alphabaculovirus—Lymantria dispar multiple nucleopolyhedrovirus (LdMNPV)—has been applied successfully to control outbreaks of the gypsy moth, another defoliating lepidopteran pest found in the northeastern USA. However, while LdMNPV can cause naturally occurring epizootics in outbreaking populations of *Lymantria dispar*, OpbuNPV appears to exist primarily in a covert state in Massachusetts winter moth populations, with little mortality reported in field-caught, laboratory-raised larvae, and no viral epizootics reported in Massachusetts populations [5,8,9]. An early attempt to control an outbreak of winter moth larvae in British Columbia with an application of OpbuNPV caused only a transient 46% reduction in larval population density with the highest dose [10].

In this study, we examined the occlusion bodies and the genome sequence of a Massachusetts isolate of OpbuNPV (OpbuNPV-MA) as part of an attempt to understand why this virus is not more of a mortality factor in North American populations of the winter moth and to identify genotypes that may be more virulent against winter moth larvae.

## 2. Materials and Methods

### 2.1. Virus Production and Isolation

A sample of the isolate OpbuNPV-MA collected in eastern Massachusetts was used to infect 3rd instar *O. brumata* larvae that had been hatched and reared in captivity as described in [9]. Larvae that were starved overnight were allowed to feed on a cube of diet surface-contaminated with 8 × 10^5^ OpbuNPV-MA OBs.

Cadavers dying from infection were harvested and homogenized in 20 mL 0.5% SDS for 30 s with an Ultra-Turrax T-25 fitted with an IKA S25N-18G dispersing tool and set at 3000 rpm. The homogenate was filtered through three layers of cheesecloth, and OBs were recovered by low-speed centrifugation (1436 *g* for 10 min). After decanting the supernatant, the OB pellet was resuspended in 25 mL 0.1% SDS. OBs were pelleted again as above, resuspended in 25 mL 0.5 M NaCl, then pelleted a third time. The final OB pellet was resuspended in 0.02% sodium azide prepared in deionized distilled H_2_O at a final concentration of 8.7 × 10^9^ OBs/mL.

### 2.2. Electron Microscopy

For scanning electron microscopy (SEM), OpbuNPV-MA OBs were pipetted onto filter paper and secured to copper plates using ultra-smooth round carbon adhesive tabs (Electron Microscopy Sciences, Inc., Hatfield, PA, USA). The OBs were frozen by placing the plates on the surface of a pre-cooled (−196 °C) brass bar whose lower half was submerged in liquid nitrogen. After 20–30 s, the holders containing the frozen samples were transferred to a Quorum PP2000 cryo-prep chamber (Quorum Technologies, East Sussex, UK) attached to an S-4700 field emission scanning electron microscope (Hitachi High Technologies America, Inc., Dallas, TX, USA). The OBs were etched to remove condensed water vapor by raising the temperature of the stage to −90 °C for 10–15 min. Following etching, the temperature of the stage inside the cryo-transfer system was lowered to −130 °C, and the OBs were coated with a 10-nm layer of platinum using a magnetron sputter head equipped with a platinum target. The specimens were transferred to a pre-cooled (−130 °C) cryostage in the low-temperature SEM (LT-SEM) for observation. An accelerating voltage of 5 kV was used to view the specimens. Images were captured using a 4pi Analysis System (Durham, NC, USA).

For transmission electron microscopy (TEM), OBs were pelleted by centrifugation at 2300× *g* for 3 min. The pellet was fixed for 2 h at room temperature in 2.5% glutaraldehyde-0.05 M sodium cacodylate-0.005 M CaCl_2_ (pH 7.0), then refrigerated at 4 °C overnight. After six rinses with 0.05 M sodium cacodylate-0.005 M CaCl_2_ buffer, the OBs were post-fixed in 1% buffered osmium tetroxide for 2 h at room temperature. Post-fixed OBs were then rinsed six times in the same buffer, dehydrated in a graded series of ethanol followed by three exchanges of propylene oxide, infiltrated in a graded series of LX-112 resin/propylene oxide, and polymerized in LX-112 resin at 65 °C for 24 h. Then, 60- to 90-nm silver-gold ultrathin sections were cut on a Reichert/AO Ultracut ultramicrotome with a Diatome diamond knife and mounted onto 200 mesh carbon/formvar-coated copper grids. Grids were stained with 4% uranyl acetate and 3% lead citrate and imaged at 80 kV with a Hitachi HT-7700 transmission electron microscope (Hitachi High Technologies America, Inc., USA).

### 2.3. Viral DNA Isolation and Sequencing

An aliquot of OpbuNPV-MA containing 6.5 × 10^9^ OBs was diluted to 28 mL in 0.1 M Na_2_CO_3_. Diluted OBs were solubilized by incubation for 30 min at the benchtop, followed by 15 min at 37 °C, and insoluble material was removed by centrifugation (10 min at 1436 *g*). After neutralization with 1 M Tris-HCl pH 7.5, ODVs were pelleted by centrifugation (75 min at 103,586 *g*) through a 3 mL pad of 25% *w*/*w* sucrose in phosphate-buffered saline using a Beckman SW-28 rotor. DNA was extracted from the ODV pellet by resuspension in Disruption Buffer (10 mM Tris-HCL pH 8.0–10 mM EDTA pH 8.0–0.25% SDS) containing 500 μg/mL proteinase K, followed by incubation for 1 h at 55 °C, extraction with 1:1 phenol:chloroform, and ethanol precipitation. A total of 0.5 μg DNA was recovered as assessed with the Quant-iT PicoGreen dsDNA Kit (Invitrogen, Waltham, MA, USA).

For sequencing of the viral DNA, a paired-end library was prepared by tagmentation of 100 ng of the DNA sample with the QIAseq FX DNA Library Kit (Qiagen catalog #180473) followed by size selection with the GeneRead Size Selection Kit (Qiagen catalog #180514) following the manufacturer’s instructions. The library prep quality was evaluated with the Agilent TapeStation. Six picomoles of the library were sequenced on a MiSeq System (Illumina) using the MiSeq^®^ Reagent Kit v2 micro 300 cycles kit (MS-103-1002) following the manufacturer’s instructions. The sequencing data were initially assembled de novo with SeqMan NGen (DNASTAR) using 200,000 reads. The resulting contigs were joined into a single contig, with the initial nucleotide corresponding to the initial adenine of the polyhedrin start codon. This contig was then used as a template for a second round of assembly using 746,754 sequence reads with an average length of 151 nt. Single-nucleotide polymorphisms (SNPs) and insertions/deletions (indels) were identified and enumerated with the SNP Report function of SeqMan Pro (DNASTAR). The sequence of the final contig, with an average coverage of 941X, was deposited in GenBank with the accession number MF614691.

### 2.4. Genome Sequence Analysis and Feature Annotation

Lasergene GeneQuest (DNAStar, v. 14) was used to identify potentially protein-encoding open reading frames (ORFs) of ≥50 codons in size (excluding the stop codon) in the OpbuNPV-MA genome sequence. These ORFs were selected for annotation if they possessed significant amino acid sequence similarity with ORFs from other baculoviruses or sequences from other sources, as assessed by BLASTp. ORFs with no significant matches to other sequences also were selected for annotation if (a) they did not overlap a larger ORF by ≥75 bp, and (b) they were predicted to be protein-encoding by both the fgenesV (http://linux1.softberry.com/berry.phtml) and ZCURVE_V [11] algorithms. Sequence similarity for these ORFs was also sought for using HHpred [12].

Regions of repeated sequences corresponding to likely homologous repeat (*hr*) regions were also sought out and identified using Lasergene GeneQuest. Unit repeats were aligned with CLUSTAL W [13]) on Lasergene MegAlign (v. 14) and conserved positions were visualized with BoxShade 3.2 (http://www.ch.embnet.org/software/BOX_form.html).

### 2.5. Sequence Comparison and Phylogeny

Amino acid sequences were aligned using MAFFT [14] on MegAlign Pro (v. 14) with default parameters. Core gene amino acid sequence alignments were concatenated using BioEdit 7.1.3.0.

Minimum evolution (ME) phylograms were inferred using MEGA 7 [15] using the Jones–Taylor–Thorton (JTT) substitution matrix with rates varying among sites and a gamma parameter value estimated from the alignments. For the ME phylogram of the concatenated core gene alignments, the DNA polymerase alignment was used to estimate the gamma parameter. The pairwise-deletion option was used for handling gaps and missing data, and tree reliability was evaluated by bootstrap with 500 replicates.

Maximum likelihood (ML) phylograms were inferred with either MEGA 7 from single-sequence alignments or RAxML [16] from the concatenated core gene alignments. For MEGA 7, the best-fitting substitution matrix—as determined by the Model Selection function of MEGA 7—was used for phylogenetic inference with variable rates among sites, the pairwise-deletion option for handling gaps and missing data, and 500 bootstrap replicates. For RAxML, the Le and Gascuel (LG) substitution matrix was used with variable rates among sites and 100 bootstrap replicates.

## 3. Results

### 3.1. Ultrastructure of OBs from Winter Moth Larvae

**Figure 1:**
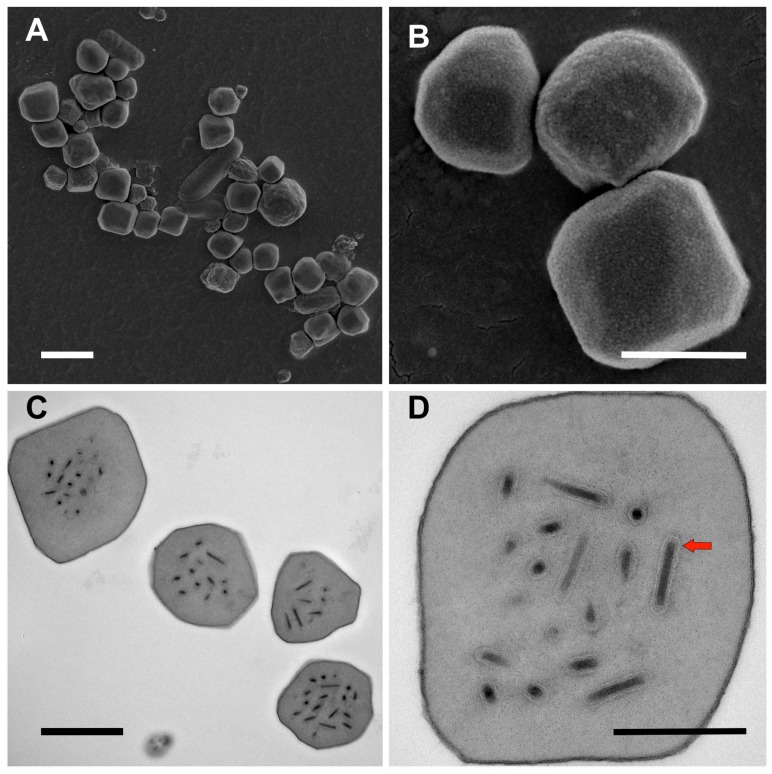
Occlusion bodies (OBs) of alphabaculovirus OpbuNPV-MA (Massachusetts isolate of OpbuNPV). (**A**,**B**) Scanning electron micrographs of OpbuNPV-MA OBs. (**C**,**D**) Transmission electron micrographs of OpbuNPV-MA OBs. The red arrow in (**D**) denotes the envelope surrounding the rod-shaped nucleocapsid of a virion. Scale bars: (**A**) 2 μm; (**B**,**C**) 1 μm; (**D**) 500 nm.

Scanning electron micrographs revealed that the OBs isolated from winter moth larval cadavers were composed of irregular polyhedra with an appearance typical for alphabaculovirus OBs (Figure 1A,B). OBs measured up to 1.8 μm in diameter. Transmission electron micrographs of sections through the OBs revealed that they contained numerous enveloped rod-shaped virions consisting of a single nucleocapsid per virion (Figure 1C,D).

**Figure 2:**
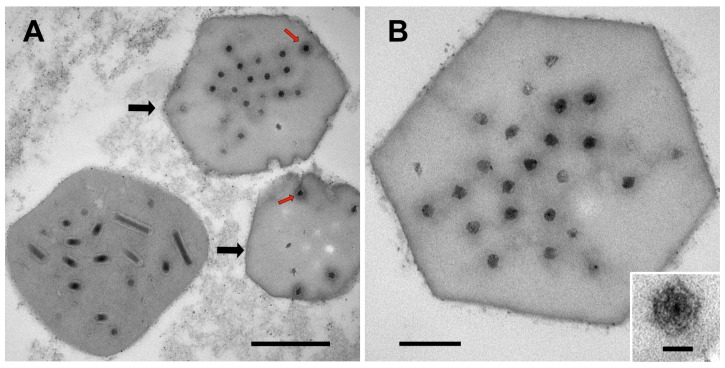
OBs of presumptive cypoviruses found in the OpbuNPV-MA OB preparation. (**A**) OpbuNPV-MA and cypovirus OBs. Black arrows denote two cypovirus OBs adjacent to an OpbuNPV-MA OB, and red arrows point to examples of the virus-like particles in the presumptive cypovirus OBs. (**B**) Higher magnification image of a presumptive cypovirus OB, with an inset showing a high magnification image of one of the occluded virus-like particles. Scale bars: (**A**) 500 nm; (**B**) 200 nm; (**inset**) 40 nm.

Closer examination of the OB cross-sections revealed the presence of OBs that did not contain the rod-shaped virions typically observed in baculovirus OBs (Figure 2A). Instead, these OBs contained virus-like particles that were round or icosahedral in cross-section and measured up to 58 nm in diameter (Figure 2B). The OBs themselves were often hexagonal in cross-section and were usually smaller relative to the alphabaculovirus OBs in the sectioned material. In appearance, the OBs together with their embedded virions closely resembled OBs described for cypoviruses, which are viruses of the insect-specific genus *Cypovirus* of the segmented dsRNA virus family Reoviridae [17]. At least two distinct cypoviruses have previously been described from Scottish populations of *O. brumata* [18,19].

### 3.2. Properties of the OpbuNPV-MA Genome Sequence

**Figure 3:**
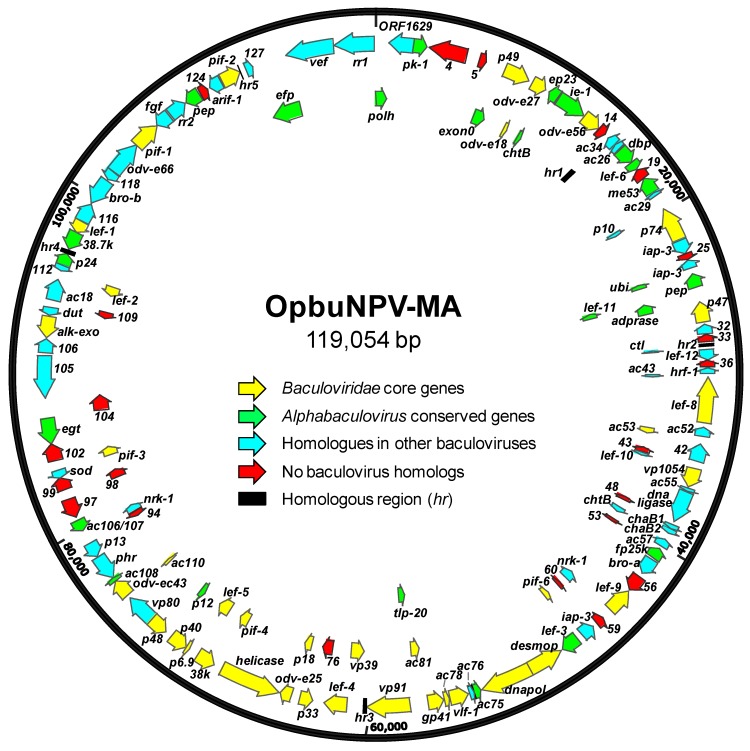
Map of the open reading frames (ORFs) and other features of the OpbuNPV-MA genome. ORFs are represented by arrows, with the position and direction of the arrow indicating ORF position and orientation. Each ORF is color-coded to indicate whether it corresponds to a baculovirus core gene, an ORF conserved among all alphabaculoviruses, an ORF with homologs in a subset of other baculoviruses, or an ORF with no baculovirus homologs. Homologous repeat regions (*hr*s) are represented by black rectangles. ORFs are designated by names if they are conserved or well-characterized baculovirus homologs, or a number corresponding to its annotation in the genome.

**Figure 4:**
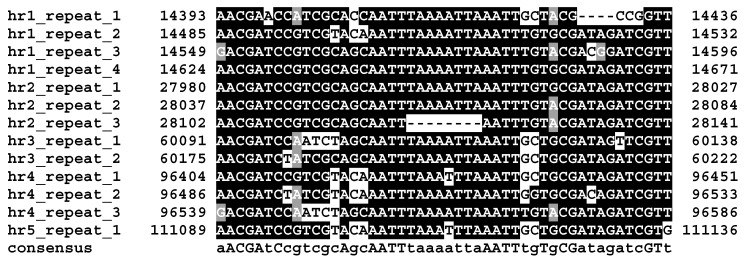
OpbuNPV-MA homologous region (*hr*) unit repeats. Nucleotide positions of the repeats in the genome sequence are indicated. Identical nucleotides occupying >50% of aligned positions are shaded in black, and nucleotides of the same class as conserved nucleotides (containing either a purine or pyrimidine base) are shaded in gray. Uppercase letters in the consensus sequence denote nucleotides in the alignment with completely identical residues, and lowercase letters denote positions in the alignment with a majority of identical residues.

Reads from sequencing of OpbuNPV-MA DNA were assembled into a circular genome contig of 119,054 bp (Figure 3). The size of the genome and the G+C nucleotide distribution (39.83%) were within the range of genome sizes and nucleotide distributions that have been reported for other alphabaculoviruses [20]. A total of 130 ORFs were annotated (Table S2). In addition, five homologous repeat regions (*hr*s) [20] were identified that contained one-to-four copies of a 48-bp imperfect inverted repeat sequence with the consensus sequence 5′-aACGAtCcgtcgcAgcAATTtaaaattaAATTtgTgCGatagatcGTt-3′ (Figure 4).

In the assembly, 878 putative SNPs and small indels were identified. These polymorphisms were present at low frequencies within the assembly, with only ten occurring at a frequency of ≥8%, and most occurring at frequencies ≤6% (Figure S1). In contrast, a sequence of an Autographa californica multiple nucleopolyhedrovirus isolate generated with an Illumina HiSeq 2000 instrument identified a frequency cluster of 118 SNPs with average frequencies of 33–36% [21]. The highest-frequency SNPs in the OpbuNPV-MA assembly occurred at nucleotide positions 1,320 (17.44%), 7,468 (14.63%), 66,442 (11.08%), 66,453 (10.10%), and 114,338 (12.5%). All five SNPs occur in ORFs, and the last four listed are nonsynonymous substitutions in ORF6 (*exon0*), ORF80 (*helicase*), and ORF129 (*vef*). In addition, reads corresponding to larger (>3 bp) indels were present in ORFs 55 (*baculovirus repeated ORF-a*, or *bro-a*) and 117 (*bro-b*) at frequencies of approximately 6%, and in other locations at lower frequencies.

### 3.3. OpbuNPV-MA ORF Content

#### 3.1.1. Core Genes, *bro* Genes, and Unique ORFs

The OpbuNPV-MA genome contains all 38 of the core genes identified to-date in every baculovirus genome, including *ac110* (*pif-8*) (Figure 3, Table S2) [22,23]. It also contains 25 of the 26 ORFs identified by Garavaglia et al. [22] as present in genomes of alpha- and betabaculoviruses, and is missing *ac64* (*gp37*). There are two copies of the *baculovirus repeated ORF* (*bro*) multigene family [24] present in the genome sequence.

Twenty-two ORFs were determined not to possess homologs in any other baculovirus genome (Figure 3, Table S2). Among these ORFs, ORF4 exhibited 56% sequence identity with part of a hypothetical protein from *Xenoplus laevis.* ORF33 encoded a sequence exhibiting 53.8% identity with an uncharacterized ORF identified in the winter moth genome (accession No. KOB64482) [25], suggesting an exchange of genetic material between virus and host during their co-evolution. Queries with HHpred using the encoded amino acid sequences indicated the presence of zinc finger domains in ORF59 and ORF60. The remaining nineteen unique ORFs did not exhibit sequence similarity with other sequences in queries with BLASTp and HHpred.

#### 3.1.2. Inhibitor-Of-Apoptosis Protein (*iap*) Genes

Three ORFs—ORF24, ORF26, and ORF61—were found to encode inhibitor-of-apoptosis protein (IAP) homologs [26]. BLASTp results suggested that all three of these ORFs encode homologs of the *iap-3* lineage. This feature is unusual, as alphabaculoviruses generally contain only one *iap* gene of any given lineage, and often possess homologs of *iap-1* and *iap-2* [26]. While all three IAP homologs contain two copies of the *Baculovirus Inhibitor of apoptosis protein Repeat* (BIR) domain, only ORFs 24 and 61 contain the C-terminal RING finger domain common to IAPs. ORF26 encodes a relatively short IAP sequence (159 amino acids), and it appears that a sequence at the C-terminus that would encode a RING finger is missing.

**Figure 5:**
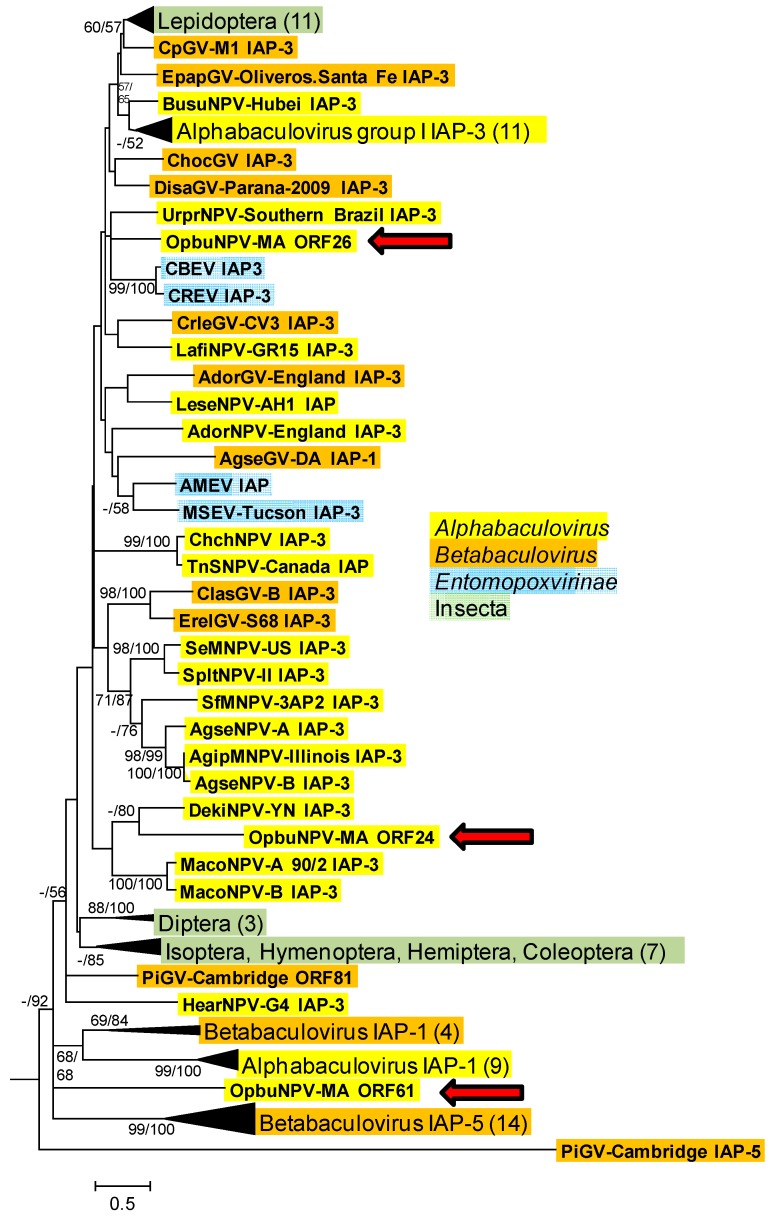
Phylogenetic inference from aligned viral and insect inhibitor-of-apoptosis protein (IAP) homologs. Maximum likelihood (ML) phylogram inferred from the alignment of IAP amino acid sequences is shown with bootstrap values (>50%) at interior branches for minimum evolution (ME) and ML analysis (ME/ML) where they occur. The sequence for *Arabadopsis thaliana* IAP-like protein (GenBank accession no. NP_173164) was used as an outgroup. The classification of each taxon is indicated with a color-coded text background. Branches for IAPs from various insect orders and for alphabaculovirus IAP-1, betabaculovirus IAP-1, betabaculovirus IAP-5, and group I alphabaculovirus IAP-3 sequences are collapsed, and the numbers of taxa in each of these nodes are indicated in parentheses. OpbuNPV sequences are indicated with red arrows. The virus and insect taxa and their accession numbers are as listed in Table S1.

Phylogenetic inference with selected baculovirus IAP-1, baculovirus IAP-3, betabaculovirus IAP-5, entomopoxvirus IAP, and insect IAP sequences did not place any of the three OpbuNPV-MA IAP sequences with any of the IAP-1, IAP-5, group II alphabaculovirus IAP-3, entomopoxvirus, or insect IAP clades in the ML phylogram (Figure 5). ORF24 was grouped with the IAP-3 of Dendrolimus kikuchii nucleopolyhedrovirus (DekiNPV-YN; GenBank accession No. JX193905) in both ME and ML phylograms, although bootstrap support >50% for this placement only occurred in the ML phylogram. ORFs 26 and 61 occurred in different parts of the ME and ML phylograms with bootstrap support <50%.

#### 3.1.3. DNA Ligase III and Host Range Factor-1 (*hrf-1*)

OpbuNPV-MA ORF37 and ORF47 were found to encode host range factor-1 (*hrf-1*) and a homolog of DNA ligase III, respectively. Both of these genes were originally identified in the genome sequence of LdMNPV isolate 5/6 [27]. The LdMNPV *hrf-1* gene allows heterologous alphabaculoviruses to replicate in the *L. dispar* Ld652Y cell line [28,29]. The LdMNPV DNA ligase III gene product has been shown to ligate nicks in a double-stranded DNA substrate, suggesting that it may play a role in viral DNA replication or repair [30]. Homologs of *hrf-1* have only been identified in LdMNPV, Dasychira pudibunda nucleopolyhedrovirus (DapuNPV) [31], and Orgyia pseudotsugata multiple nucleopolyhedrovirus (OpMNPV) [32], while baculovirus homologs of DNA ligase III have been found only in LdMNPV, Lymantria xylina nucleopolyhedrovirus (LyxyMNPV-5) [33], Sucra jujuba nucleopolyhedrovirus (SujuNPV) [34], and Orgyia leucostigma nucleopolyhedrovirus (OrleNPV) [35].

While the LdMNPV and LyxyMNPV-5 DNA ligase sequences share significant sequence identity, BLASTp queries with any of the other baculovirus DNA ligase III sequences returned matches with insect DNA ligases and not baculovirus homologs, suggesting that baculovirus DNA ligase III homologs are not closely related to each other and may have been independently acquired by their respective viruses.

#### 3.1.4. *chaB* (*ac58/59* and *ac60*) Genes

**Figure 6:**
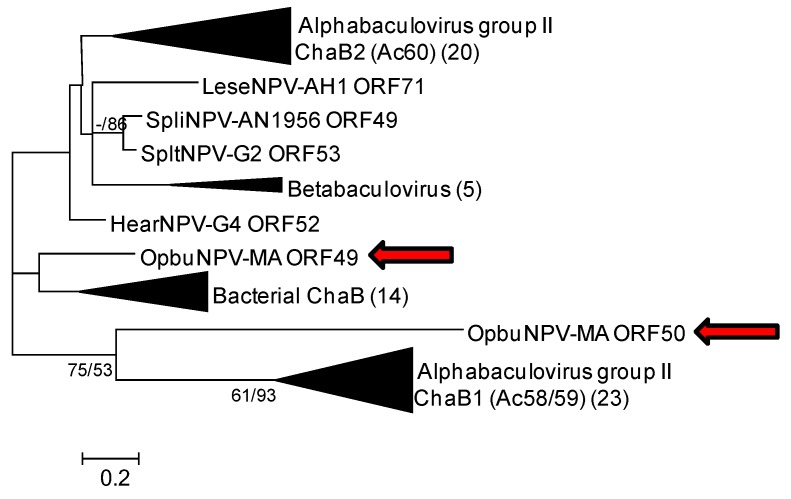
Phylogenetic inference from aligned viral and bacterial cation transporter (ChaB) homologs. An ML phylogram inferred from the alignment of ChaB amino acid sequences is shown with bootstrap values (>50%) at interior branches for ME and ML analysis (ME/ML) where they occur. Branches for ChaB sequences encoded by bacteria, betabaculoviruses, alphabaculovirus *chaB1* (*ac58/59*) genes, and alphabaculovirus *chaB2* (*ac60*) genes have been collapsed, and the numbers of taxa in each of these nodes are indicated in parentheses. OpbuNPV sequences are indicated with red arrows. The virus and bacterial taxa and their accession numbers are as listed in Table S1.

Alphabaculoviruses and some betabaculoviruses contain homologs of the bacterial ion transport regulator gene *chaB* [36,37]. These homologs often occur as an adjacent pair of ORFs that correspond to AcMNPV ORFs *ac58/59* (*chaB1*) and *ac60* (*chaB2*). The OpbuNPV-MA genome sequence also contains two adjacent *chaB* homologs encoded by ORF49 and ORF50, but a BLASTp search with ORF49 yielded matches that consisted mostly of bacterial *chaB* gene products with few matches to baculovirus *chaB* sequences. In ME and ML phylograms, separate clusters were formed that contained the betabaculovirus *chaB* homologs, the alphabaculovirus *ac58/59* homologs, the alphabaculovirus *ac60* homologs, and the bacterial *chaB* genes (Figure 6). ORF49 was positioned as a sister taxon to the bacterial *chaB* clade in the ML phylogram, and was placed within the bacterial clade in the ME phylogram, but neither arrangement enjoyed >50% bootstrap support. ORF50 was placed with the *ac58/59* sequences in a basal position.

#### 3.1.5. Two ORFs with Sequence Similarity to Nicotinamide Riboside Kinase 1 (*nrk1*)

BLASTp queries with OpbuNPV-MA ORF95 returned several matches with group II alphabaculovirus NRK1 sequences, suggesting that this ORF encodes a baculovirus nicotinamide riboside kinase 1 homolog. Although ORF58 also exhibits sequence similarity with baculovirus NRK1 sequences by BLAST, the top BLASTp matches for ORF58 are betabaculovirus sequences. There is no significant sequence similarity between the ORF58 and ORF95 amino acid sequences.

Other alphabaculovirus *nrk1* ORFs encode polypeptides that are approximately 360 amino acids, while the sequence encoded by ORF95 is only 159 amino acids. The ORF95 sequence aligns with the C-terminal end of other baculovirus NRK1 sequences—a region which contains a sequence matching a number of different phosphatase domains. In contrast, ORF58 appears to be a full-length homolog of the *nrk1*-like ORFs found in betabaculovirus genomes. Cellular NRK1 is involved in the biosynthesis of nicotinamide adenine dinucleotide (NAD+) [38,39], but the function of the baculovirus NRK1 homologs is unclear.

#### 3.1.6. DNA Photolyase (*phr*)

**Figure 7:**
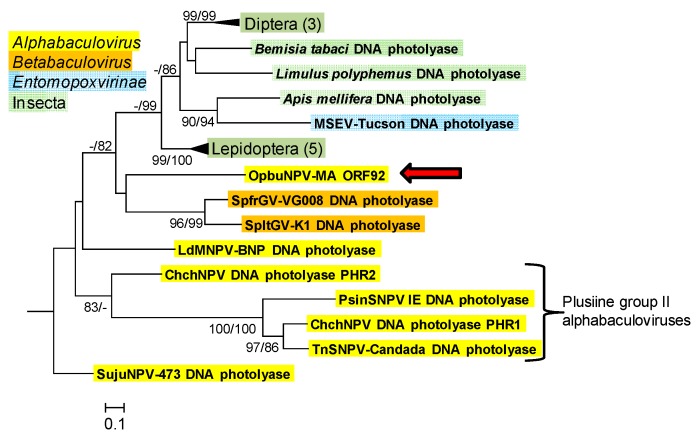
Phylogenetic inference from aligned viral and insect DNA photolyase homologs. An ML phylogram inferred from the alignment of DNA photolyase amino acid sequences is shown with bootstrap values (>50%) at interior branches for ME and ML analysis (ME/ML) where they occur. The sequence for *Bacillus cereus* ATCC 14579 DNA photolyase (GenBank accession No. AAP10079) was included as an outgroup. The classification of each taxon is indicated with a color-coded text background. Branches for DNA photolyases from the insect orders Diptera and Lepidoptera are collapsed, and the numbers of taxa in each of these nodes are indicated in parentheses. The OpbuNPV sequence is indicated with a red arrow, and sequences from alphabaculoviruses of the lepidopteran subfamily Plusiinae are indicated with a bracket. The virus and insect taxa and their accession numbers are as listed in Table S1.

ORF92 of the OpbuNPV-MA genome encodes a protein with significant sequence similarity to DNA photolyases from a variety of insect sources and a DNA photolyase homolog from Spodoptera frugiperda granulovirus (SpfrGV-VG008) [40]. A small number of photolyase sequences have been identified from alpha- and betabaculoviruses [34,41,42,43], but only one of these homologs—PHR2 from Chrysodeixis chalcites nucleopolyhedrovirus (ChchNPV)—has been demonstrated to be capable of catalyzing the repair of UV-induced cyclobutane pyrimidine dimers [44]. Phylograms inferred from an alignment of baculovirus and insect photolyase amino acid sequences support previous findings that photolyase sequences from plusiine baculoviruses occur as a monophyletic group [45], but baculovirus sequences as a whole did not form a monophyletic group in this analysis (Figure 7). The OpbuNPV-MA photolyase sequence grouped with sequences from *Spodoptera* sp. betabaculoviruses in both ME and ML phylograms, but bootstrap support for this position was <50%.

#### 3.1.7. Ribonucleotide Reductase Large Subunit (*rr1*), Small Subunit (*rr2*), and dUTPase (*dut*)

Ribonucleotide reductase and dUTPase are cellular enzymes involved in the biosynthesis of deoxynucleotides [46,47]. Large DNA viruses often encode homologs of these enzymes [48,49]. To date, ORFs encoding homologs of dUTPase (*dut*) and the large and small subunits of ribonucleotide reductase (*rr1* and *rr2*, respectively) have been identified in isolates of 23 alphabaculovirus species and 8 betabaculovirus species. Most of these viruses encode homologs for both the ribonucleotide reductase subunits and the dUTPase, while some encode either the ribonucleotide reductase subunits or the dUTPase. ORFs encoding all three homologs—*rr1*, *rr2*, and *dut*—were found in the OpbuNPV-MA sequence.

BLASTp queries with the OpbuNPV-MA dUTPase sequence encoded by ORF108 did not return any matches with other baculovirus dUTPase homologs. The lack of significant sequence similarity with other baculovirus *dut* ORFs has been observed with other baculovirus *dut* genes [50]. Prior phylogenetic analyses of baculovirus DUT sequences have indicated that the *dut* genes in baculoviruses are the consequence of multiple gene acquisition events [51,52], and it is possible that the OpbuNPV-MA *dut* sequence also derives from an independent instance of horizontal gene transfer.

The amino acid sequence encoded by OpbuNPV-MA *rr1* (ORF130) also returned no matches with baculovirus *rr1* homologs when used in a BLASTp search. Only a single baculovirus *rr2* gene (LdMNPV-BNP *rr2b*) occurred among the matches found with a search using OpbuNPV-MA *rr2* (ORF122). For both OpbuNPV-MA subunit ORFs, the matches consisted of sequences from a variety of viral and cellular sources.

**Figure 8:**
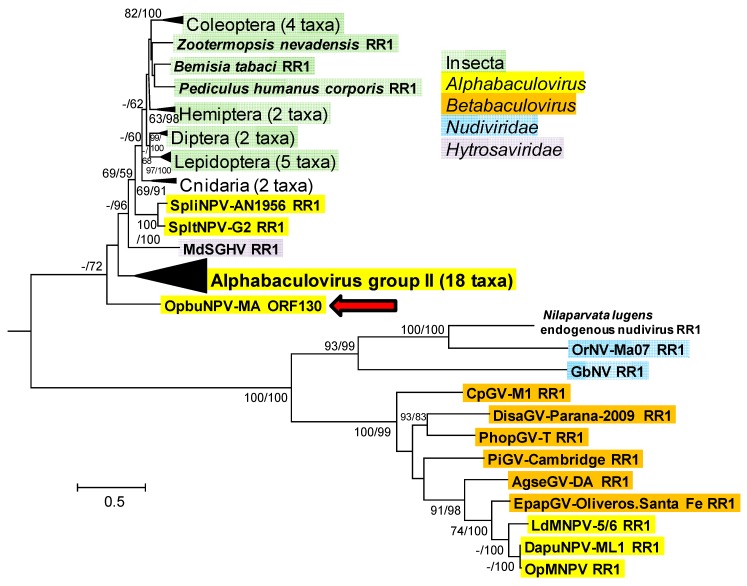
Phylogenetic inference from aligned viral and cellular ribonucleotide reductase large subunit (RR1) homologs. An ML phylogram inferred from the alignment of RR1 amino acid sequences is shown with bootstrap values (>50%) at interior branches for ME and ML analysis (ME/ML) where they occur. The sequence for *Bacillus thuringiensis* IBL200 RR1 (GenBank accession no. EEM97377) was included as an outgroup. The classification of each taxon is indicated with a color-coded text background, except for unique taxa of phylum Cnidaria and the *Nilaparvata lugens* endogenous nudivirus (which is not actually classified as a nudivirus). Branches for RR1 sequences from phylum Cnidaria; the insect orders Coleoptera, Diptera, Hemiptera, and Lepidoptera, and group II alphabaculoviruses are collapsed, and the numbers of taxa in each of these nodes are indicated in parentheses. The OpbuNPV sequence is indicated with a red arrow. The sequences in this tree and their accession numbers are as listed in Table S1.

In phylograms of ORF130 (RR1) and other selected RR1 sequences, the taxa occurred in one group consisting of nudivirus, alphabaculovirus, and betabaculovirus sequences, and a second group consisting of OpbuNPV-MA ORF130, a clade containing most of the alphabaculovirus RR1 sequences in the tree, and a collection of alphabaculovirus, hytrosavirus, insect, and cnidiarians (Figure 8). OpbuNPV-MA ORF130 occupied a basal position relative to all the other sequences in the second group in both the ME and ML phylograms, although bootstrap support for this position was >50% only in the ML phylogram.

**Figure 9:**
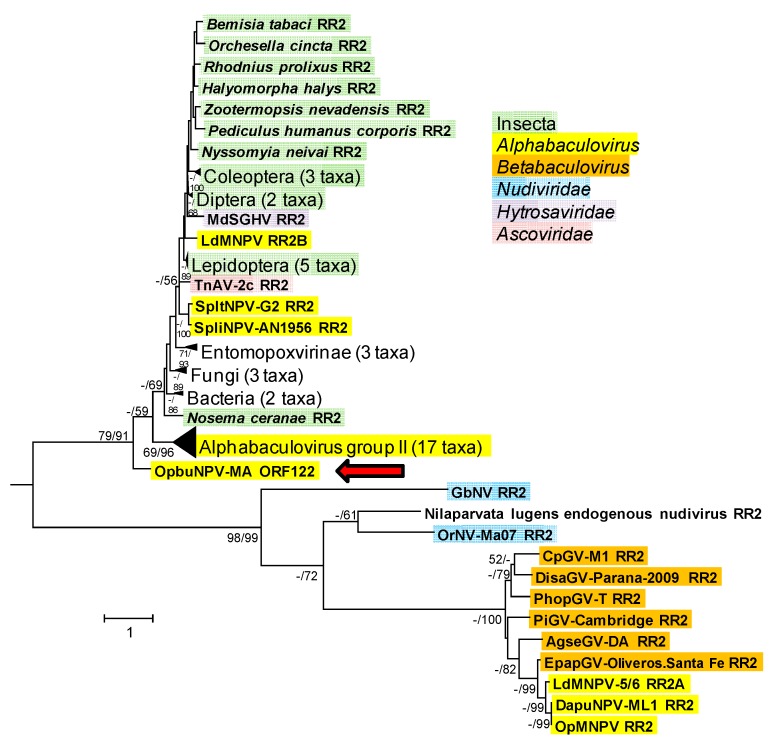
Phylogenetic inference from aligned viral and cellular ribonucleotide reductase small subunit (RR2) homologs. An ML phylogram inferred from the alignment of RR1 amino acid sequences is shown with bootstrap values (>50%) at interior branches for ME and ML analysis (ME/ML) where they occur. The sequence for *Bacillus thuringiensis* IBL200 RR2 (GenBank accession no. EEM97378) was included as an outgroup. The classification of each taxon is indicated with a color-coded text background, except for unique taxa of subfamily *Entomopoxvirinae*, kingdoms Fungi and Bacteria, and the *Nilaparvata lugens* endogenous nudivirus (which is not actually classified as a nudivirus). Branches for RR1 sequences from the entomopoxviruses, the fungi, the bacteria, the insect orders Coleoptera, Diptera, and Lepidoptera, and group II alphabaculoviruses are collapsed, and the numbers of taxa in each of these nodes are indicated in parentheses. The OpbuNPV sequence is indicated with a red arrow. The sequences in this tree and their accession numbers are as listed in Table S1.

Phylogenetic inference with ORF122 (RR2) and other selected RR2 sequences produced trees with a topology that was strikingly similar to the RR1 tree (Figure 9). The RR2 ME and ML phylograms also consisted of a group with nudivirus, alphabaculovirus, and betabaculovirus sequences and a group consisting of a clade containing most of the alphabaculovirus sequences and a collection of RR2 sequences from cellular and other viral sources. The OpbuNPV-MA RR2 sequence was placed in the same basal position in the second group occupied by OpbuNPV-MA RR1 in the RR1 phylograms, with bootstrap support >50% for both trees. In both the RR1 and RR2 trees, the sequences from LdMNPV-5/6, DapuNPV-ML1, and OpMNPV were grouped with the betabaculovirus and nudivirus sequences, while other alphabaculovirus sequences (including the second LdMNPV-5/6 RR2 sequence, RR2B) occurred in the second group.

### 3.4. Relationship of OpbuNPV-MA to Other Baculoviruses

The OpbuNPV-MA polyhedrin (*polh*) nucleotide sequence from the genome shared 99.2% and 99.0% with the partial *polh* sequences of OpbuNPV-MA1 (GenBank accession No. HQ663848) and OpbuNPV-UK1 (HQ663847) previously generated by Burand et al. [8]. Additionally, the OpbuNPV-MA *p74* sequence from the genome shared 100% sequence identity with the OpbuNPV-MA partial *p74* sequence generated by Broadley et al. [9]. These results indicate that the OpbuNPV preparations from Massachusetts winter moth populations and an OpbuNPV geographic isolate from the United Kingdom are variants of the same virus sequenced in this study.

A previous analysis of the relationships of OpbuNPV-MA with other baculoviruses, based on phylogenetic inference from nucleotide alignments of the *polyhedrin* (*polh*) and *p74* genes, placed OpbuNPV-MA among group II alphabaculoviruses [9]. The absence of a gene encoding the GP64 budded virus envelope protein also denotes OpbuNPV-MA as a group II alphabaculovirus. The top matches from BLASTp queries with OpbuNPV-MA ORFs encoding baculovirus homologs consisted mostly of a wide variety of group II alphabaculoviruses, but did not indicate a particularly close relationship with any specific virus. The sequence identities between 97 conserved baculovirus homologs in OpbuNPV-MA (excluding BRO and P6.9 sequences) and their top matches by BLASTp ranged from 24.6% (LEF-3) to 82.9% (polyhedrin), with a median sequence identity of 44.5%. The pairwise sequence identities for 37 core gene homologs in OpbuNPV-MA (excluding P6.9) ranged from 31.9% (DNA helicase) to 74.1% (LEF-9), with a median sequence identity of 50.5%.

**Figure 10:**
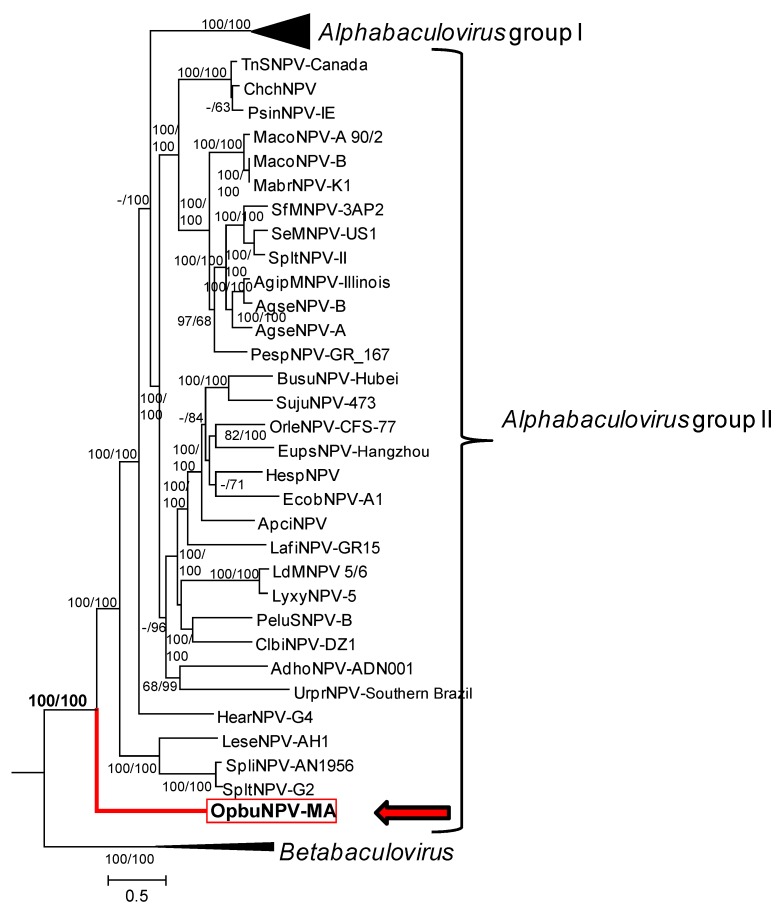
Relationships of OpbuNPV-MA and representative isolates of other baculovirus species, inferred from the predicted amino acid sequences of baculovirus core genes. The ML phylogram was constructed from the concatenated alignments of 38 baculovirus core gene amino acid sequences using the ME method. Shown is a subtree of a tree that also included viruses of the genera *Gammabaculovirus* and *Deltabaculovirus.* Branches for betabaculoviruses and the group I alphabaculoviruses are collapsed, and the numbers of taxa in those nodes are shown in parentheses. Group II alphabaculoviruses are indicated with brackets. Bootstrap values >50% for both ME and ML analysis are indicated for each interior branch (ME/ML). OpbuNPV-MA is indicated with a red arrow, red branch, and red box surrounding the taxon. The taxa and sequences used in the analysis are as listed in Table S1.

Phylogenetic inference from the concatenated alignments of the 38 core gene amino acid sequences placed OpbuNPV-MA in a clade with other alphabaculoviruses, but in a basal position relative to the group II alphabaculoviruses (Figure 10). This placement was strongly supported in both ME and ML phylograms. The separation of group I and group II alphabaculoviruses into distinct monophyletic clades was also confirmed by this analysis.

## 4. Discussion

Two cypoviruses—Operophtera brumata cypovirus 18 (OpbuCPV18) and Operophtera brumata cypovirus 19 (OpbuCPV19)—were discovered along with a non-occluded reovirus present in both *O. brumata* larvae and an associated parasitoid in winter moth populations in the Orkney Isles north of Scotland and characterized by Graham and coworkers [18,19]. OpbuCPV and OpbuNPV were detected in the same larval cadavers, with a frequency of co-occurrence that varied among the populations sampled and ranged from 0% to 70.8% [18]. The OpbuCPV isolates also were found to be capable of vertical transmission. Given the above, it is possible that the presumptive OpbuCPV OBs discovered in an OB preparation from the Massachusetts OpbuNPV-killed cadavers were carried over by winter moths that invaded North America from Europe. Sequencing of the RNA segments in the Massachusetts larval OB preparation will determine whether the Massachusetts larvae are carrying OpbuCPV18 and OpbuCPV19, or a different cypovirus. Such knowledge will also allow for screening of North American populations of winter moth for the presence and frequency of cypovirus infections.

While there have been other reports of the co-occurrence of cypoviruses and baculoviruses [53,54], there are few studies documenting the interaction of these two groups of viruses during co-infection. In a book chapter on cypoviruses, Belloncik and Mori [55] cite a publication in a French language journal and a conference presentation purporting to show a synergistic effect on mortality of insects infected with both cypoviruses and baculoviruses. They also allude to unpublished results of tissue culture co-infections that suggest interference between the two types of viruses [55]. The most comprehensive study on the interaction of cypoviruses and baculoviruses examined the results of infection of two lepidopteran hosts (*Choristoneura fumiferana* and *Malacosoma disstria*), with matched pairs of baculoviruses and cypoviruses from these hosts [56]. With both hosts and their baculovirus/cypovirus pairs, prior infection with the cypovirus was found to interfere with a subsequent infection with the baculovirus, retarding the development of nuclear polyhedrosis. Dependent on how prevalent cypoviruses are among North American populations, interference by a cypovirus may explain observations of a lack of OpbuNPV pathogenicity against North American winter moth larvae.

One purpose for sequencing the OpbuNPV-MA genome was to assess the genetic variability that could be sampled to identify genotypes that—either singly or in combination—would exhibit greater pathogenicity against winter moth larvae. The assembled sequence reads of the genome revealed a low level of genetic variation in the OpbuNPV-MA genome, such that isolating different genotypes from OpbuNPV-MA to test in bioassays would be technically challenging. In contrast, a significant degree of genetic variation was detected in populations of winter moth in its native habitat in Scotland by restriction endonuclease analysis [7]. The low level of genetic variation observed in OpbuNPV-MA may be due to a population bottleneck [57]. The low percentages of polymorphism abundance in the OpbuNPV-MA genome sequence assembly is consistent with a narrow bottleneck and a very low number of founder genotypes—possibly only a single founder genotype. The potential of producing single-genotype baculovirus populations from low-dose infections has been demonstrated in laboratory studies [58,59]. It is not known if the low genetic variability is specific to the stock used to sequence OpbuNPV-MA. If so, it suggests that a bottleneck event occurred during production of the stock. Alternatively, if the lack of variability is discovered to be a feature of OpbuNPV populations in Massachusetts winter moth larvae, then the bottleneck event may have occurred during the invasion of Massachusetts by the winter moth. PCR and sequencing with primers designed to amplify a loci known to be variable among baculovirus isolates (for example, the *bro* genes) should provide more information on the extent to which the low variability reported for the OpbuNPV-MA genome sequence is present in other virus populations.

Assuming that the low degree of variability is widespread among other populations of OpbuNPV in Massachusetts, it may also be a factor in the apparent lack of pathogenicity observed with this virus. Other studies have found that infections with mixtures of genotypes cause more mortality than single-genotype infections [60,61]. Such observations suggest that the OpbuNPV-MA isolate may be missing genotypes that are required for optimal levels of pathogenicity.

Analysis of the OpbuNPV-MA ORFs indicates that it represents a divergent lineage among the group II alphabaculoviruses. Phylogenetic inference with core gene amino acid sequence alignments placed OpbuNPV-MA on a branch by itself and basal to the clade containing other alphabaculovirus taxa. These results suggests that the lineage leading to OpbuNPV appeared early during the evolution and diversification of the alphabaculoviruses. Sequence data from the alphabaculovirus of the related moth *Operopthera bruceata*—a native North American geometrid species—suggests that the *O. bruceata* nucleopolyhedrovirus (OpbrNPV) is also part of this lineage [9].

## 5. Conclusions

Examination of the OBs of OpbuNPV-MA and determination of its genome sequence have pointed to potential explanations for why OpbuNPV-MA is not more of a mortality factor among North American populations of the winter moth. It is anticipated that further research on cypoviruses and other isolates of alphabaculoviruses of the winter moth and related species should increase our understanding of factors affecting the development of viral disease in winter moth populations and expand our insight into the evolution of baculoviruses. In addition, genome sequences of OpbuNPV from other locations may yield more information on the genetic variability normally present among populations of this virus.

## Supplementary Materials

The following are available online at www.mdpi.com/1999-4915/9/10/307/s1. Figure S1: Frequencies of polymorphisms in the OpbuNPV-MA genome assembly; Table S1: Names, abbreviations, and GenBank accession numbers of taxa used in phylogenetic inference; Table S2: OpbuNPV-MA open reading frames (ORFs) and homologous repeat regions (*hr*s).
