# Supplementary material for: The Operophtera brumata Nucleopolyhedrovirus (OpbuNPV) Represents an Early, Divergent Lineage within Genus Alphabaculovirus
Source: Viruses. 2017 Oct 21;9(10):307. doi: 10.3390/v9100307 (PMC5691658; doi:10.3390/v9100307)
Supplement: Supplementary file 1 [file viruses-09-00307-s001.zip › Figure S1 - Frequencies of polymorphisms in the OpbuNPV-MA genome assembly.pptx]

## Slide 1
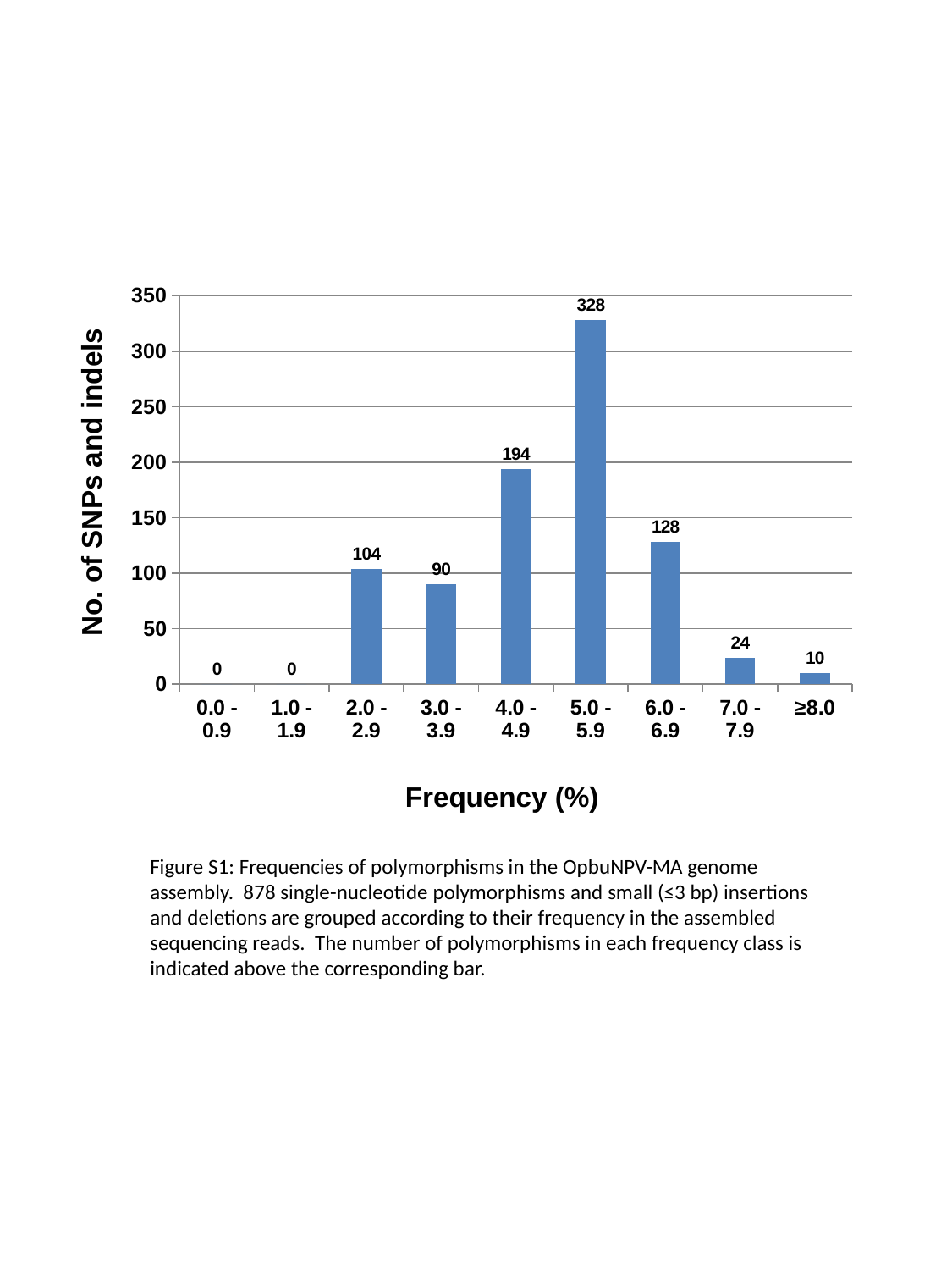

### Chart
| Category | |
|---|---|
| 0.0 - 0.9 | 0.0 |
| 1.0 - 1.9 | 0.0 |
| 2.0 - 2.9 | 104.0 |
| 3.0 - 3.9 | 90.0 |
| 4.0 - 4.9 | 194.0 |
| 5.0 - 5.9 | 328.0 |
| 6.0 - 6.9 | 128.0 |
| 7.0 - 7.9 | 24.0 |
| ≥8.0 | 10.0 |No. of SNPs and indels
Frequency (%)
Figure S1: Frequencies of polymorphisms in the OpbuNPV-MA genome assembly. 878 single-nucleotide polymorphisms and small (≤3 bp) insertions and deletions are grouped according to their frequency in the assembled sequencing reads. The number of polymorphisms in each frequency class is indicated above the corresponding bar.
