# Supplementary material for: The Operophtera brumata Nucleopolyhedrovirus (OpbuNPV) Represents an Early, Divergent Lineage within Genus Alphabaculovirus
Source: Viruses. 2017 Oct 21;9(10):307. doi: 10.3390/v9100307 (PMC5691658; doi:10.3390/v9100307)
Supplement: Supplementary file 1 [file viruses-09-00307-s001.zip › Table S2 OpbuNPV-MA open reading frames (ORFs) and homologous repeat regions (hrs).docx]

**Table S2.** OpbuNPV-MA open reading frames (ORFs) and homologous repeat regions (*hr*s)

| **ORF** | **Name** | **Position** | **aa** | **Top BLASTp match^1^** | **% identity (range of alignment)** | **Notes/AcMNPV homologs^2^** |
| --- | --- | --- | --- | --- | --- | --- |
| 1 | *polh* | 1🡪741 | 246 | Polyhedrin [Adoxophyes honmai nucleopolyhedrovirus] | 82.9% (204/246) | *ac8* |
| 2 | *orf1629* | 738🡨2141 | 467 | predicted wiskott-Aldrich syndrome protein family member 1 [*Stunus vulgaris*] | 32.0% (39/122) | *ac9*  Drf_FH3 (pfam06367), prob=98.7% (aa 21-81); WH2 (pfam02205), prob=87.9% (aa 343-370) |
| 3 | *pk1* | 2143🡪2940 | 265 | Protein kinase-1[Agrotis segetum nucleopolyhedrovirus B] | 38.2% (99/259) | *ac10* |
| 4 |  | 3043🡨5250 | 735 | hypothetical protein XENTR_v90025753mg [*Xenopus tropicalis*] | 56% (61/109) | cell division protein ZipA (prk03427), e=2.63e-05 (aa 378-492) |
| 5 |  | 5990🡪6406 | 138 | NSS |  |  |
| 6 | *exon0* | 6815🡪7585 | 256 | exon-0 [Chrysodeixis chalcites nucleopolyhedrovirus] | 34.5% (78/226) | *ac141* |
| 7 | *p49* | 7582🡪9099 | 505 | hypothetical protein [Spodoptera litura nucleopolyhedrovirus] | 48% (223/465) | *ac142* |
| 8 | *odv-e18* | 9059🡪9310 | 83 | ODV-E18[Leucania separata nucleopolyhedrovirus] | 51.2% (43/84) | *ac143* |
| 9 | *odv-e27* | 9319🡪10164 | 281 | ODV-E27 [Spodoptera litura nucleopolyhedrovirus] | 38.7% (106/274) | *ac144* |
| 10 | *chtB-like* | 10149🡪10427 | 92 | ChtB1 [Buzura suppressaria nucleopolyhedrovirus] | 58.7% (54/92) | *ac145* |
| 11 | *ep23* | 10419🡨11009 | 196 | early 23 kDa protein [Orgyia leucostigma NPV] | 31.4% (59/188) | *ac146* |
| 12 | *ie-1* | 11050🡪12792 | 580 | IE1 [Hemileuca sp. nucleopolyhedrovirus] | 24.9% (97/390) | *ac147* |
| 13 | *odv-e56* | 12862🡪13926 | 354 | envelope protein [Lymantria dispar multiple nucleopolyhedrovirus] | 51.3% (178/347) | *ac148* |
| 14 |  | 13954🡪14394 | 146 | NSS |  |  |
|  | *hr1* | 14393-14671 |  |  |  | 4 repeats |
| 15 |  | 14680🡨15219 | 179 | Hypothetical protein SlsnVgp016 [Spodoptera littoralis nucleopolyhedrovirus] | 45.4% (84/185) | *ac34* |
| 16 |  | 15569🡨15228 | 113 | unknown [Spodoptera litura nucleopolyhedrovirus] | 36.5% (42/115) | *ac26* |
| 17 | *dbp* | 15650🡪16588 | 312 | DNA binding protein [Helicoverpa armigera nucleopolyhedrovirus] | 31.8% (101/318) | *ac25* |
| 18 | *lef6* | 16641🡪17087 | 148 | LEF-6 [Lymantria dispar multiple nucleopolyhedrovirus] | 42.4% (28/66) | *ac28* |
| 19 |  | 17165🡨17755 | 196 | NSS |  |  |
| 20 | *me53* | 17806🡨18744 | 312 | me53 [Leucania separata nucleopolyhedrovirus] | 34% (110/324) | *ac139* |
| 21 |  | 18783🡨18986 | 67 | hypothetical protein [Thysanoplusia orichalcea nucleopolyhedrovirus] | 35.8% (24/67) | *ac29* |
| 22 | *p10* | 19617🡪19856 | 79 | p10 [Agrotis segetum nucleopolyhedrovirus B] | 71.4% (50/70) | *ac137* |
| 23 | *p74* | 19853🡨21799 | 648 | p74 [Euproctis pseudoconspersa nucleopolyhedrovirus] | 57.8% (381/659) | *ac138* |
| 24 |  | 21864🡪22682 | 272 | inhibitor of apoptosis 3 [Diatraea saccharalis granulovirus] | 35.2% (92/261) |  |
| 25 |  | 22721🡪23050 | 109 | NSS |  |  |
| 26 |  | 23108🡨23584 | 158 | inhibitor of apoptosis protein isoform X1 [Bombyx mori] | 50.0% (99/198) | Also - IAP3 [Hyphantria cunea nucleopolyhedrovirus], e=2.1e-51, and other baculovirus IAP3s |
| 27 |  | 23811🡨23581 | 76 | ubiquitin-60S ribosomal protein L40, putative [Plasmodium gallinaceum] | 85.5% (65/76) | *ac35* |
| 28 | *pp31* | 23920🡨24714 | 264 | 39 kDa protein [Leucania separata nucleopolyhedrovirus] | 33.7% (86/255) | *ac36* |
| 29 | *lef-11* | 24575🡨24991 | 138 | LEF-11 [Leucania separata nucleopolyhedrovirus] | 44.7% (55/123) | *ac37* |
| 30 | *bv-e31; nudix* | 24922🡨25593 | 223 | hypothetical protein [Helicoverpa armigera nucleopolyhedrovirus] | 54% (108/200) | *ac38* |
| 31 | *p47* | 25590🡨26771 | 393 | protein p47 [Lymantria dispar multiple nucleopolyhedrovirus] | 60.1% (235/391) | *ac40* |
| 32 |  | 26852🡨27430 | 192 | hypothetical protein [Mamestra configurata nucleopolyhedrovirus A] | 30.2% (59/195) |  |
| 33 |  | 27474🡨27890 | 138 | Uncharacterized protein OBRU01_16454 [*Operophtera brumata*] | 53.8% (77/143) |  |
|  | *hr2* | 27979🡨28141 |  |  |  | 3 repeats |
| 34 | *ctl* | 28140🡨28298 | 52 | CTL [Choristoneura murinana alphabaculovirus] | 67.3% (35/52) | *ac3* |
| 35 | *lef-12* | 28341🡪28925 | 194 | LEF12 [Euproctis pseudoconspersa nucleopolyhedrovirus] | 29.8% (62/208) | *ac41* |
| 36 |  | 28951🡨29322 | 123 | NSS |  |  |
| 37 | *hrf-1* | 29364🡨29711 | 115 | host range factor 1 [Lymantria dispar multiple nucleopolyhedrovirus] | 34.7% (41/118) |  |
| 38 |  | 29721🡪29915 | 64 | hypothetical protein [Mamestra configurata nucleopolyhedrovirus B] | 46.6% (27/58) | *ac43* |
| 39 | *lef-8* | 29905🡨32523 | 872 | agip130 [Agrotis ipsilon multiple nucleopolyhedrovirus] | 66.1% (581/879) | *ac50* |
| 40 |  | 32809🡨33291 | 160 | ac52-like protein [Peridroma alphabaculovirus] | 30.1% (47/156) | *ac52* |
| 41 | *ac53* | 33394🡪33786 | 130 | hypothetical protein CaLGV115 [Clostera anastomosis granulovirus] | 49.1% (29/59) | *ac53* |
| 42 |  | 33783🡨34748 | 321 | hypothetical protein HanGV4gp044 [Helicoverpa armigera nucleopolyhedrovirus] | 31.1% (41/132) |  |
| 43 |  | 34748🡨35092 | 114 | NSS |  |  |
| 44 | *lef-10* | 35101🡪35319 | 72 | LEF-10 [Helicoverpa armigera SNPV] | 54.4% (37/68) | *ac53a* |
| 45 | *vp1054* | 35183🡪36268 | 361 | VP1054 [Buzura suppressaria nucleopolyhedrovirus] | 40.1% (142/354) | *ac54* |
| 46 |  | 36370🡪36549 | 59 | ORF-41 [Buzura suppressaria nucleopolyhedrovirus] | 35.1% (20/57) | *ac55* |
| 47 | *dna ligase* | 36588🡪38429 | 613 | CLUMA_CG017821, isoform A [Clunio marinus] | 38.3% (240/627) | Several matches to insect DNA ligase 3. |
| 48 |  | 38369🡨38623 | 84 | NSS |  |  |
| 49 | *chaB2* | 38630🡪38875 | 81 | cation transporter [Bacillus mediterraneensis] | 50.7% (34/67) |  |
| 50 | *chaB1* | 38887🡪39153 | 88 | hypothetical protein SlsnVgp048 [Spodoptera littoralis nucleopolyhedrovirus] | 32% (24/75) |  |
| 51 | *chtB-like* | 39162🡪39548 | 128 | hypothetical protein [Lymantria dispar multiple nucleopolyhedrovirus] | 37.3% (25/67) | *ac150* |
| 52 |  | 40000🡨39542 | 152 | hypothetical protein [Choristoneura rosaceana alphabaculovirus] | 36.8% (57/155) | *ac57* |
| 53 |  | 40057🡪40263 | 68 | NSS |  |  |
| 54 | *fp25k* | 40818🡨40195 | 207 | few polyhedra protein [Spodoptera litura nucleopolyhedrovirus] | 61.1% (121/198) | *ac61* |
| 55 | *bro-a* | 40952🡪41929 | 325 | BRO [Trichoplusia ni granulovirus LBIV-12] | 52.8% (188/356) |  |
| 56 |  | 42143🡪43081 | 312 | NSS |  |  |
| 57 | *lef-9* | 44704🡨43175 | 509 | late expression factor 9 [Helicoverpa armigera nucleopolyhedrovirus] | 74.1% (363/490) | *ac62* |
| 58 | *nrk-1-like* | 45287🡨44691 | 198 | hypothetical protein [Diatraea saccharalis granulovirus] | 46.3% (87/188) |  |
| 59 |  | 45761🡨45306 | 151 | PREDICTED: myotubularin-related protein 8 isoform X2 [Cimex lectularius]; e = 0.043 | 50% (14/28) | zf-RING_5 (pfam14634), prob=95.3% (aa 46-93) |
| 60 |  | 45997🡨45758 | 79 | zinc finger protein 518A [Castor canadensis]; e=0.04 | 33.8% (24/71) | zf-H2C2_5 (PF13909), prob=95.5% (aa 28-50) |
| 61 |  | 46048🡨46824 | 258 | inhibitor of apoptosis 3 [Urbanus proteus nucleopolyhedrovirus] | 30.4% (80/263) |  |
| 62 | *pif-6* | 46809🡨47192 | 127 | hypothetical protein SlsnVgp060 [Spodoptera littoralis nucleopolyhedrovirus] | 53.2% (66/124) | *ac68* |
| 63 | *lef-3* | 47191🡪48234 | 347 | LEF-3 [Lymantria dispar multiple nucleopolyhedrovirus] | 24.6% (93/378) | *ac67* |
| 64 | *desmop* | 48292🡨50403 | 703 | orf417 [Helicoverpa zea single nucleopolyhedrovirus] | 43% (46/107) | *ac66* |
| 65 | *dnapol* | 50405🡪53389 | 994 | DNAPOL [Leucania separata nucleopolyhedrovirus] | 46.9% (478/1020) | *ac65* |
| 66 |  | 53853🡨53437 | 138 | ac75 [Malacosoma sp. alphabaculovirus] | 28.5% (39/137) | *ac75* |
| 67 |  | 54115🡨53858 | 85 | hypothetical protein [Lymantria dispar multiple nucleopolyhedrovirus] | 55.3% (47/85) | *ac76* |
| 68 | *vlf-1* | 54164🡨55297 | 377 | VLF-1 [Spodoptera littoralis nucleopolyhedrovirus] | 72% (278/386) | *ac77* |
| 69 |  | 55305🡨55610 | 101 | unknown [Spodoptera frugiperda multiple nucleopolyhedrovirus] | 32.7% (36/110) | *ac78* |
| 70 | *gp41* | 56574🡨55615 | 319 | glycoprotein GP41 [Spodoptera littoralis nucleopolyhedrovirus] | 52.9% (162/306) | *ac80* |
| 71 | *ac81* | 57160🡨56558 | 200 | hypothetical protein [Orgyia leucostigma NPV] | 62.3% (119/191) | *ac81* |
| 72 | *tlp-20* | 57602🡨57093 | 169 | TLP-20 [Sucra jujuba nucleopolyhedrovirus] | 46.8% (65/139) | *ac82* |
| 73 | *vp91* | 57571🡪60090 | 839 | VP91 CAPSID [Agrotis segetum nucleopolyhedrovirus] | 44.9% (368/820) | *ac83* |
|  | *hr3* | 60091-60222 |  |  |  | 2 repeats |
| 74 | *vp39* | 60298🡨61188 | 296 | VP39 capsid [Spodoptera litura nucleopolyhedrovirus] | 34% (100/294) | *ac89* |
| 75 | *lef-4* | 61187🡪62518 | 443 | LEF-4 [Agrotis segetum nucleopolyhedrovirus] | 48.9% (224/458) | *ac90* |
| 76 |  | 62523🡪63194 | 223 | NSS |  |  |
| 77 | *p33* | 63938🡨63180 | 252 | hypothetical protein, partial [Helicoverpa armigera nucleopolyhedrovirus] | 53% (134/253) | *ac92* |
| 78 | *p18* | 63940🡪64410 | 156 | ORF-82 [Agrotis segetum nucleopolyhedrovirus] | 59.7% (92/154) | *ac93* |
| 79 | *odv-e25* | 64407🡪65090 | 227 | occlusion-derived virus envelope protein ODV-E25 [Spodoptera litura nucleopolyhedrovirus] | 62.6%  (142/227) | *ac94* |
| 80 | *helicase* | 65145🡨68708 | 1187 | helicase [Leucania separata nucleopolyhedrovirus] | 31.9% (404/1267) | *ac95* |
| 81 | *pif-4* | 68536🡪69189 | 217 | hypothetical protein SlnV2_gp074 [Spodoptera litura nucleopolyhedrovirus II] | 60.6% (100/165) | *ac96* |
| 82 | *38k* | 69186🡨70262 | 358 | ORF103 [Leucania separata nucleopolyhedrovirus] | 47.3% (166/351) | *ac98* |
| 83 | *lef-5* | 70062🡪70847 | 261 | Late expression factor 5 lef-5 [Spodoptera exigua multiple nucleopolyhedrovirus] | 57.4% (143/249) | *ac99* |
| 84 | *p6.9* | 71056🡨70844 | 70 | NSS |  | *ac100;* Arg/Ser-rich protein with conserved position adjacent to *lef-5* |
| 85 | *p40* | 72079🡨71081 | 332 | hypothetical protein [Spodoptera litura nucleopolyhedrovirus] | 41% (137/334) | *ac101* |
| 86 | *p12* | 72470🡨72105 | 121 | P12 [Hyphantria cunea nucleopolyhedrovirus] | 33.6% (36/107) | *ac102* |
| 87 | *p48* | 73591🡨72470 | 373 | hypothetical protein SlsnVgp088 [Spodoptera littoralis nucleopolyhedrovirus] | 61.6% (228/370) | *ac103* |
| 88 | *vp80* | 73610🡪75370 | 586 | VP80 [Agrotis ipsilon multiple nucleopolyhedrovirus] | 28.6% (36/126) | *ac104* |
| 89 |  | 75367🡪75537 | 56 | ac110 [Hemileuca sp. nucleopolyhedrovirus] | 55.6% (30/54) | *ac110* |
| 90 | *odv-ec43* | 75545🡪76606 | 353 | hypothetical protein AhnVgp082 [Adoxophyes honmai nucleopolyhedrovirus] | 50.1% (181/361) | *ac109* |
| 91 |  | 76613🡪76828 | 71 | hypothetical protein [Spodoptera litura nucleopolyhedrovirus] | 44.6% (33/74) | *ac108* |
| 92 | *phr* | 76863🡨78305 | 480 | PREDICTED: deoxyribodipyrimidine photo-lyase-like [Papilio xuthus] | 53.3% (240/450) |  |
| 93 | *p13* | 78361🡨79230 | 289 | ORF99 [Helicoverpa SNPV AC53] | 60% (162/270) |  |
| 94 |  | 79543🡨79190 | 117 | NSS |  |  |
| 95 | *nrk-1* | 79600🡪80076 | 158 | nicotinamide riboside kinase 1 [Apocheima cinerarium nucleopolyhedrovirus] | 39.3% (66/168) |  |
| 96 |  | 80701🡨80066 | 211 | hypothetical protein [Chrysodeixis includens nucleopolyhedrovirus] | 66.8% (151/226) | *ac106/107* |
| 97 |  | 80878🡨82017 | 379 | NSS |  |  |
| 98 |  | 82209🡪82766 | 185 | NSS |  |  |
| 99 |  | 82597🡪83298 | 233 | NSS |  |  |
| 100 | *sod* | 83762🡨83304 | 152 | SOD [Chrysodeixis chalcites nucleopolyhedrovirus] | 77.2% (115/149) | *ac31* |
| 101 | *pif-3* | 83761🡪84366 | 201 | hypothetical protein [Helicoverpa armigera nucleopolyhedrovirus] | 47% (94/200) | *ac115* |
| 102 |  | 84363🡪85289 | 308 | NSS |  |  |
| 103 | *egt* | 85316🡨86788 | 490 | ecdysteroid UDP-glucosyltransferase [Apocheima cinerarium nucleopolyhedrovirus] | 48.9% (228/466) | *ac15* |
| 104 |  | 86880🡪87932 | 350 | NSS |  |  |
| 105 |  | 90385🡨87929 | 818 | Unknown (Ld129) [Spodoptera exigua multiple nucleopolyhedrovirus] | 26.7% (191/716) |  |
| 106 |  | 90575🡪91360 | 261 | hypothetical protein [Chrysodeixis includens nucleopolyhedrovirus] | 31.7% (65/205) | Far-17a_AIG1 (pfam04750), prob=99.9% (aa 55-249) |
| 107 | *alk-exo* | 91427🡨92662 | 411 | alkaline exonuclease [Helicoverpa armigera nucleopolyhedrovirus] | 44.5% (186/418) | *ac133* |
| 108 | *dut* | 92727🡨93158 | 143 | deoxyuridine 5'-triphosphate nucleotidohydrolase, mitochondrial precursor, putative [*Entamoeba histolytica* HM-3:IMSS]; | 46.3% (62/134) |  |
| 109 |  | 93160🡨93597 | 145 | NSS |  |  |
| 110 |  | 93596🡪94819 | 407 | hypothetical protein EONV_gp092 [Ectropis obliqua nucleopolyhedrovirus] | 29.7% (116/391) | *ac18* |
| 111 | *lef-2* | 95411🡨94773 | 212 | LEF-2 [Clanis bilineata nucleopolyhedrovirus] | 43.1% (90/209) | *ac6* |
| 112 |  | 95661🡨95350 | 103 | Se11-like protein [Clanis bilineata nucleopolyhedrovirus] | 44.4% (24/54) |  |
| 113 | *p24* | 95702🡪96403 | 233 | p24 [Hemileuca sp. nucleopolyhedrovirus] | 36.7% (87/237) | *ac129* |
|  | *hr4* | 96404-96586 |  |  |  | 3 repeats |
| 114 | *38.7k* | 96625🡨97707 | 360 | 38.7K protein [Agrotis segetum nucleopolyhedrovirus B] | 34% (119/350) | *ac13* |
| 115 | *lef-1* | 98390🡨97719 | 223 | LEF-1 [Leucania separata nucleopolyhedrovirus] | 48.6% (108/222) | *ac14* |
| 116 |  | 98417🡪99544 | 375 | hypothetical protein [Pseudoplusia includens SNPV IE] | 40.5% (151/373) | . |
| 117 | *bro-b* | 101160🡨99574 | 528 | BRO-B [Chrysodeixis includens nucleopolyhedrovirus] | 39.6% (208/525) |  |
| 118 |  | 101753🡨101223 | 176 | hypothetical protein [Chrysodeixis includens nucleopolyhedrovirus] | 31.9% (61/191) |  |
| 119 | *odv-e66* | 101795🡪103786 | 663 | PlxyGVORF30 protein [Plutella xylostella granulovirus] | 55.8% (351/629) | *ac46* |
| 120 | *pif-1* | 103797🡪105374 | 525 | hypothetical protein [Adoxophyes orana nucleopolyhedrovirus] | 50.5% (259/513) | *ac119* |
| 121 | *fgf* | 106282🡨105404 | 292 | fibroblast growth factor [Antheraea pernyi nucleopolyhedrovirus] | 39.7% (48/121) | *ac32* |
| 122 | *rr2* | 106378🡪107457 | 359 | ribonucleoside diphosphate reductase small subunit, RR2 [Musca domestica salivary gland hypertrophy virus] | 52.5% (169/322) |  |
| 123 | *pep; pp34* | 107486🡨108406 | 306 | PP34, Calyx, Polyhedron envelope protein [Perigonia lusca single nucleopolyhedrovirus] | 42% (129/307) | *ac131* |
| 124 |  | 108496🡪108963 | 155 | NSS |  |  |
| 125 |  | 109858🡨109077 | 260 | ARIF-1 [Chrysodeixis chalcites nucleopolyhedrovirus] | 32.6% (59/181) | *ac20/21* |
| 126 | *pif-2* | 109918🡪111060 | 380 | PIF-2 [Malacosoma sp. alphabaculovirus] | 67.4% (254/377) | *ac22* |
|  | *hr5* | 111089🡨111136 |  |  |  | 1 repeat |
| 127 |  | 111410🡪111832 | 140 | ORF5 [Agrotis segetum granulovirus] | 39% (53/136) |  |
| 128 | *efp* | 111855🡨113846 | 663 | EFP [Leucania separata nucleopolyhedrovirus] | 35.1% (232/661) | *ac23* |
| 129 | *vef* | 113836🡨116631 | 931 | viral enhancing factor [Lonomia obliqua multiple nucleopolyhedrovirus] | 38.3% (284/741) |  |
| 130 | *rr1* | 116650🡨118977 | 775 | Ribonucleoside-diphosphate reductase large chain, putative [*Pediculus humanus corporis*] | 58.1% (454/781) | No baculovirus matches |

^1^ NSS: No sequence similarity detected by BLASTp or HHpred query; ^2^ E-values (e) are from BLASTp queries; true-positive probabilities (prob) are from queries with HHpred
